# Insertion of a Postoperative Drain After Surgeries for Intracranial Abscesses or Empyemas—A Single-Center Retrospective Study

**DOI:** 10.3390/neurolint18090174

**Published:** 2026-09-15

**Authors:** Harun Asoglu, Tim Lampmann, Saif-Eldin Abedellatif, Mohammed Jaber, Haitham Alenezi, Mohammed Banat, Hartmut Vatter, Motaz Hamed

**Affiliations:** Department of Neurosurgery, University Hospital Bonn, Venusberg-Campus 1, 53127 Bonn, Germany

**Keywords:** abscess, empyema, intracranial drain, infection

## Abstract

Introduction: The routine insertion of a postoperative intracranial drain following surgical evacuation of brain abscesses or subdural empyemas is practiced by some surgeons to facilitate pus evacuation and to enable cavity lavage, but robust evidence for clinical benefit is lacking. This study evaluates whether postoperative drain placement affects reoperation rates, length of hospital stay, and duration of antibiotic therapy. Methods: We performed a retrospective review of consecutive adult patients who underwent surgical drainage or excision of a primary intracranial abscess or empyema at our tertiary center between January 2020 and January 2026. Patients were grouped by whether a postoperative intracranial drain was placed. The primary endpoint was recurrence requiring reoperation. Secondary endpoints included hospital length of stay, total duration of antibiotic therapy, and operative time. Continuous variables were compared with t-tests and categorical variables with Fisher’s exact test; *p* < 0.05 was considered statistically significant. Results: Eighty procedures were identified, and 36 patients met inclusion criteria for final analysis; eight (22.2%) received a postoperative intracranial drain, and 28 (77.8%) did not. Overall, 13 patients (36.1%) required reoperation for radiological or clinical progression. Reoperation occurred in 50% of patients with a drain and 32.1% without a drain (*p* = 0.422). Median hospital stay and duration of antibiotic therapy did not differ significantly between groups (median hospital stay 27.1 vs. 23.0 days, *p* = 0.50; median antibiotic duration 67.3 vs. 51.7 days, *p* = 0.445). Operative time and infection volume rates were also similar between cohorts. Conclusions: In this single-center retrospective cohort, placement of a postoperative intracranial drain after surgical management of brain abscesses or empyemas was not associated with reduced reoperation rates, shorter hospitalization, or shorter antibiotic treatment. Given the absence of demonstrable clinical benefit and the potential risks related to drain placement, routine insertion of postoperative drains cannot be recommended.

## 1. Introduction

The surgical treatment of intracranial abscess or empyema remains a clinical challenge in modern neurosurgery despite advances in anti-infective therapy. Intracranial infections, such as intracranial abscesses and subdural empyemas, are rare but life-threatening diseases, which, if untreated, can lead to rapid neurological deterioration, increased intracranial pressure, and fatal outcomes [1,2]. These conditions are associated with high morbidity and mortality [1,3]. Especially, subdural empyemas are characterized by rapid expansion within the subdural space due to the lack of anatomical barriers [4,5]. The pathogenesis is mainly related to local infection, such as sinusitis or otitis media, and can also be caused by hematogenous spread, posttraumatic injuries, or postoperative complications [1,6]. Intracranial abscesses are defined as a localized, encapsulated infection of the brain, which originates by hematogenous or contiguous spread [1]. Epidemiologic data show that intracranial abscesses are rare, but are still associated with high lethality and complication rates [7,8]. At the same time, intracranial abscesses are the main focal intracranial infections where a bacterial cause can be detected [9,10]. Other sources, such as parasitic infectious diseases, are also possible. In some cases, when antibiotic treatment is started prior to surgery, microbiological pathogens are harder to detect. Usually, the treatment is based on a multimodal therapy with surgical treatment followed by targeted antibiotic treatment [1,11]. The current guideline emphasizes surgical treatment via stereotactic aspiration, burr-hole drainage, or craniotomy [12,13]. Retrospective studies show a success rate of 80% for surgical treatment via aspiration or craniotomy [14]. Especially in lesions over 2–2.5 cm in diameter, surgical treatment is recommended [1,11]. The surgical intervention fulfills three main aspects. First, it reduces intracranial pressure. Second, it enables microbiological diagnostic confirmation. Third, it reduces the infectious load [3,11,12,13].

The role of intracranial drain placement is still a highly discussed topic. In many centers, stereotactic aspiration combined with intracranial drain placement has been established as a minimally invasive treatment option [12,15]. Especially abscesses in deeper or functionally eloquent regions are usually treated by stereotactic aspiration [12,15,16,17]. This technique not only allows for the evacuation of pus but also enables repetitive or continuous drainage, which reduces the volume and makes follow-ups easier [12,16,17]. At the same time, drain placement may introduce additional risks, including misplacement, hemorrhage, dislocation, tract formation, and secondary infection [14,18]. Modern navigation-based procedures enable precise placement of intracranial drains and reduce the risk of intracranial damage [19,20]. Additionally, in the last couple of years, the experimental use of continuous irrigation systems and the intracavitary application of antibiotics has become more important, especially in multilocular or therapy-refractory abscesses [15].

The choice of surgical treatment depends on the size, location, stage of maturation, and the clinical condition of the patient [1,12]. Intracranial abscesses in deeper or functionally eloquent regions are treated by stereotactic aspiration, whereas superficial or well-encapsulated abscesses can be completely removed through craniotomy [21,22]. Despite substantial progress in image acquisition, antibiotic therapy, and surgical treatment, the optimal strategy is still under debate in current studies [1,15]. The integration of neurosurgical, infectiological, and radiological treatment is seen as a crucial multidisciplinary approach to improve patient outcomes [14,23]. Given this context, systematic analysis of intracranial drain use as a surgical treatment is becoming increasingly relevant. The effectiveness of an intracranial drain is still unclear.

In our facility, the placement of an intracranial drain depends on the surgeon’s preference. Against this background, systematic evaluation of postoperative drainage is clinically important. The present study examined whether placement of a postoperative intracranial drain influences reoperation rates, length of hospital stay, and duration of antibiotic therapy after surgical treatment of intracranial abscesses and empyemas.

## 2. Materials and Methods

Consecutive patients who underwent surgical treatment for a primary intracranial abscess or empyema at the authors’ institution between January 2020 and January 2026 were systematically identified and included in this study. In addition to this primary criterion, several further inclusion criteria had to be fulfilled for patients to be eligible for analysis. These included an age greater than 18 years at the time of treatment, microbiologically confirmed pathogen detection, the availability of sufficient follow-up data, as well as a complete and well-documented medical history. Patients who failed to meet one or more of these predefined inclusion criteria were excluded from the final analysis to ensure data quality and consistency. Other exclusion criteria were secondary infections after surgical treatment of any intracranial process and parasitic infections. All relevant clinical and demographic data were collected retrospectively from medical records and institutional databases.

For the purpose of comparative analysis, patients were dichotomized into two distinct groups. The first group consisted of patients who received placement of an intracranial drain during surgical treatment, while the second group included patients who did not undergo intracranial drain placement. The primary outcome measure of the study was the rate of recurrence requiring reoperation. In addition to this main endpoint, several secondary outcome parameters were evaluated, including the total length of hospital stay, the duration of administered antibiotic therapy, and the overall duration of the surgical procedure. Furthermore, patients were stratified based on the presence or absence of an intracranial drain in order to allow for subgroup analyses and more detailed comparisons between the two cohorts. The follow-ups were conducted six and twelve weeks after surgery. The follow-ups continued until the antibiotic treatment could be stopped due to radiological absence of infectious tissue and absence of inflammatory markers. Out of an initial cohort of 80 patients identified during the study period, a total of 36 patients fulfilled all inclusion criteria and were therefore included in the final statistical analysis. The intracranial drain was placed under view with the help of neuronavigation. Antibiotics were not applied in the cavity. The decision to remove the intracranial drain depended on whether pus was discharged. The volumetric measurements were conducted on MRI images within 24 h prior to surgical treatment. The borders were defined by the intake of contrast agent in MRI.

Statistical analyses were conducted using SPSS Statistics software (Version 29, IBM Corp., Armonk, NY, USA). For the evaluation of continuous, not normally distributed variables, the Mann–Whitney U test was applied. In contrast, categorical variables were analyzed using Fisher’s exact test, which is particularly suitable for smaller sample sizes. A *p*-value of less than 0.05 was defined as the threshold for statistical significance in all performed analyses.

This paper complies with the STROBE checklist.

## 3. Results

**Figure 1:**
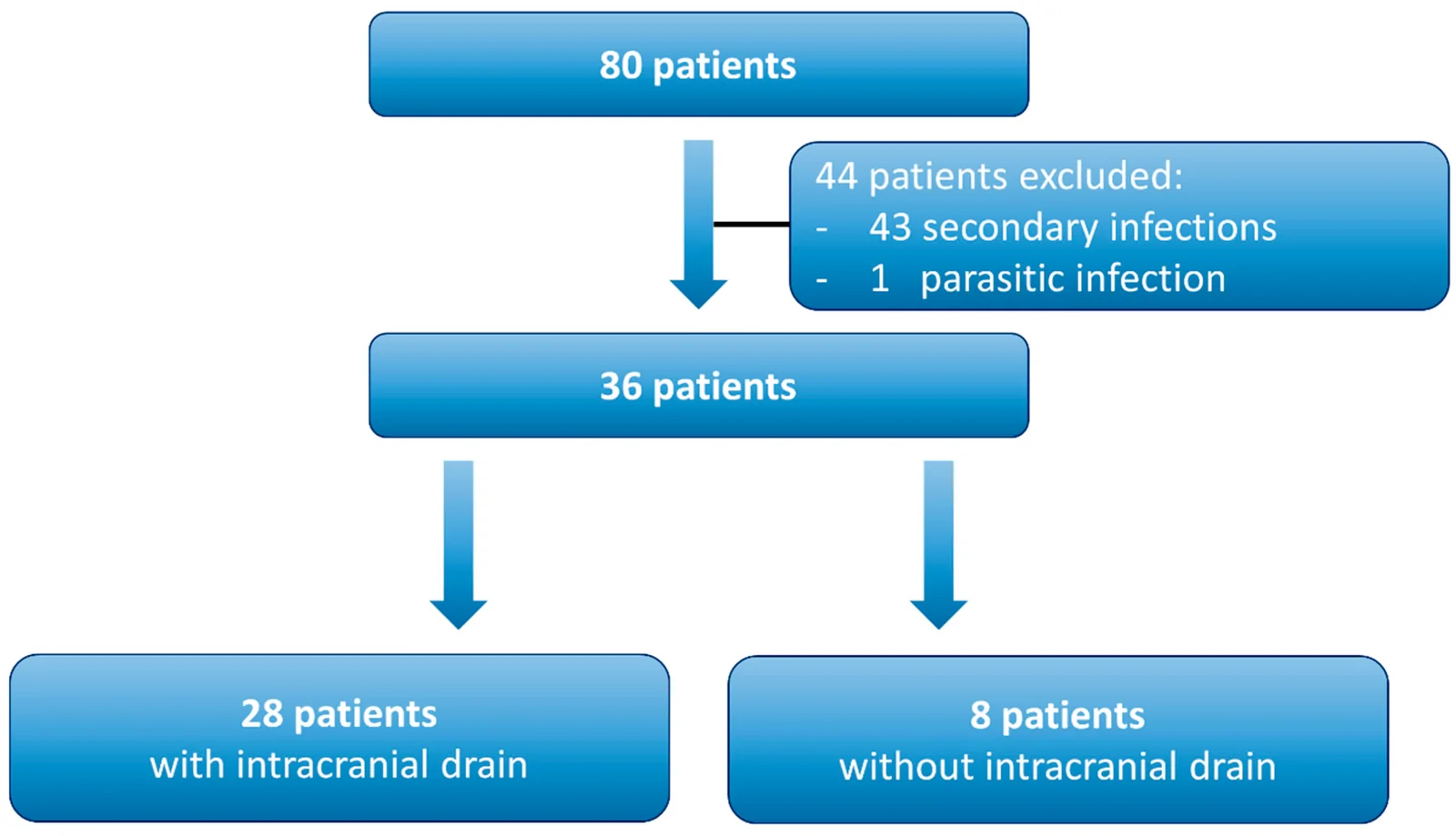
Flow chart illustrating cohort acquisition.

Figure 1 shows the flow chart for the analyzed patients with surgically treated intracranial infections between January 2020 and January 2026. The main reason for patient exclusion was the treatment of secondary infections after intracranial surgery of any kind. In our cohort, 36 patients underwent surgical treatment for a primary intracranial infection (Figure 1). Of these, 28 patients (77.8%) did not receive an intracranial drain, whereas eight patients (22.2%) underwent intracranial drain placement. Among the patients with an intracranial drain, seven were treated via burr hole and one via craniotomy. The median age of patients with an intracranial drain was 47 years, whereas the median age of patients who did not receive an intracranial drain was 60 years. Overall, 11 patients were female, and 25 were male (Table 1). Table 1 shows the comparison of groups with and without intracranial drain. No significant differences could be detected.

**Figure 2:**
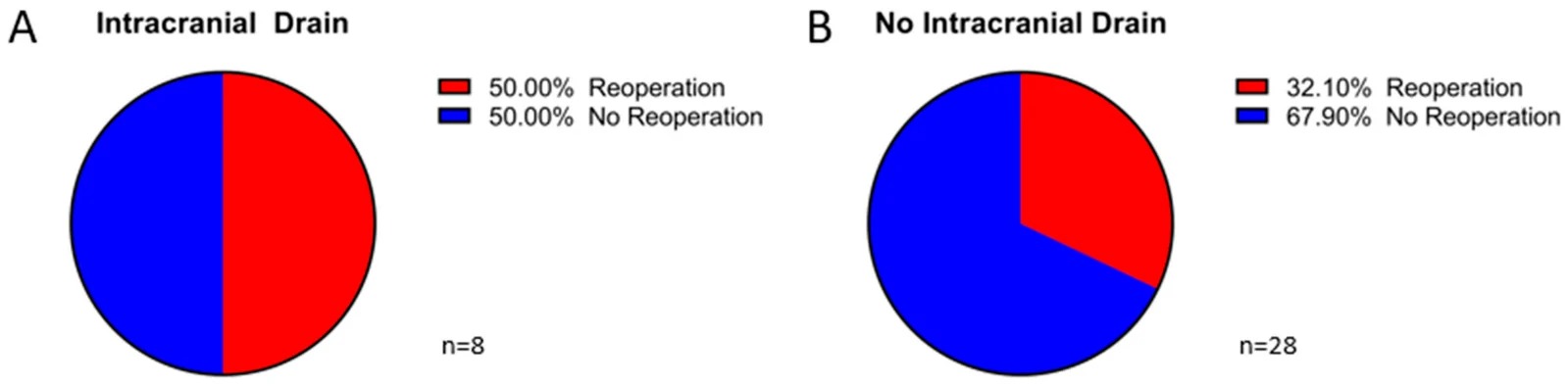
Pie charts illustrating reoperation rates in patients (**A**) with an intracranial drain and (**B**) without an intracranial drain following surgery.

Figure 2 presents pie charts illustrating reoperation rates. Panel A shows patients with an intracranial drain, of whom 50% required reoperation because of radiological progression of infectious tissue or clinical deterioration. Panel B depicts patients without an intracranial drain, with a reoperation rate of 32.1%. The difference in reoperation rates between the two groups was not significant (*p* = 0.422). Most patients underwent reoperation within 2 weeks after primary surgery (Table 1).

Table 2 shows the comparison of approach to reoperation rates. There was no significant difference between the different approaches in reoperation rates.

**Figure 3:**
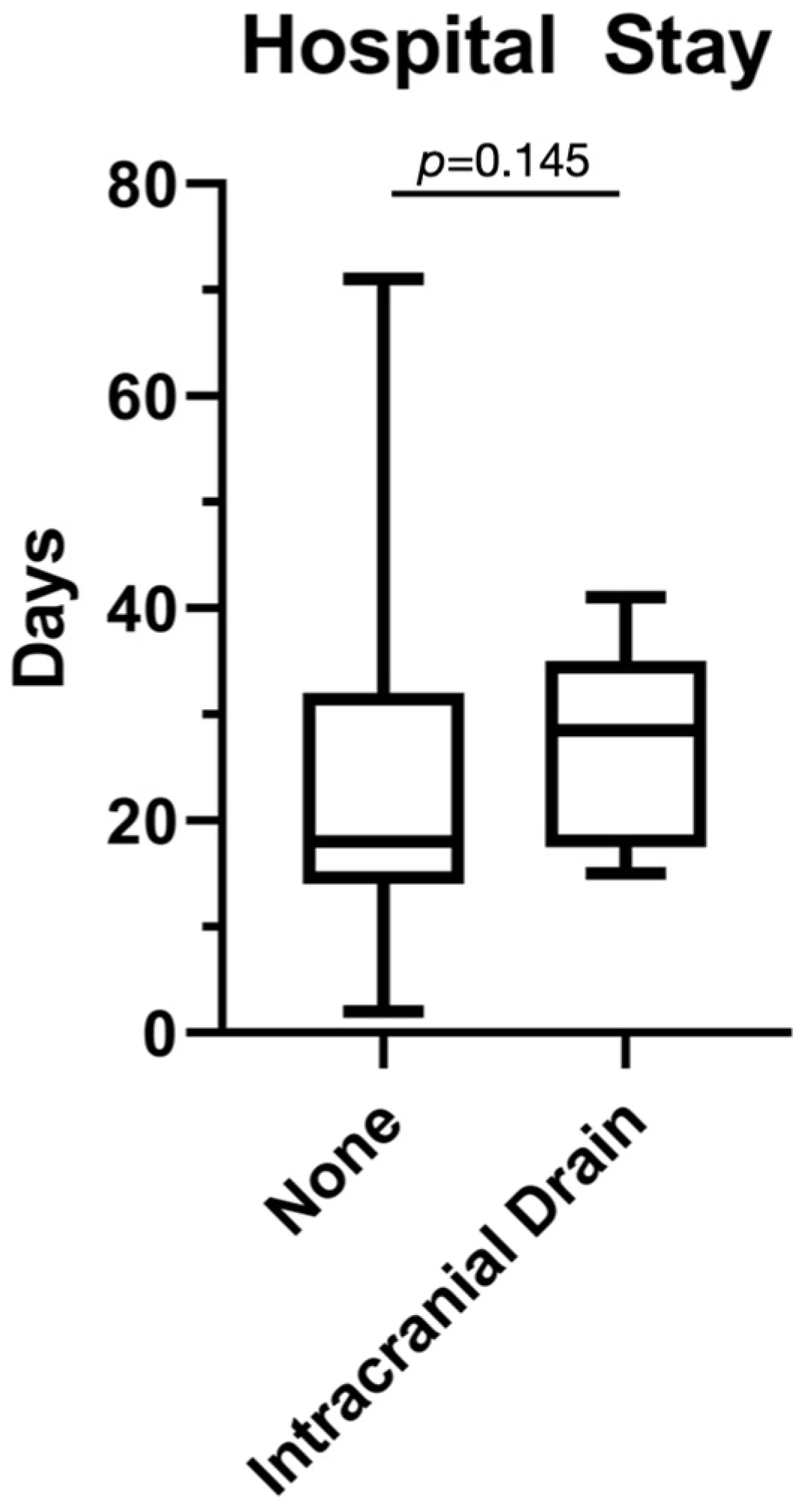
Boxplot illustrating the length of hospital stay (in days) in patients with and without an intracranial drain.

Length of hospital stay is presented in the boxplot in Figure 3. The median duration of hospitalization was 29 days in patients with an intracranial drain compared to 18 days in those without a drain; however, this difference was not significant (*p* = 0.145).

**Figure 4:**
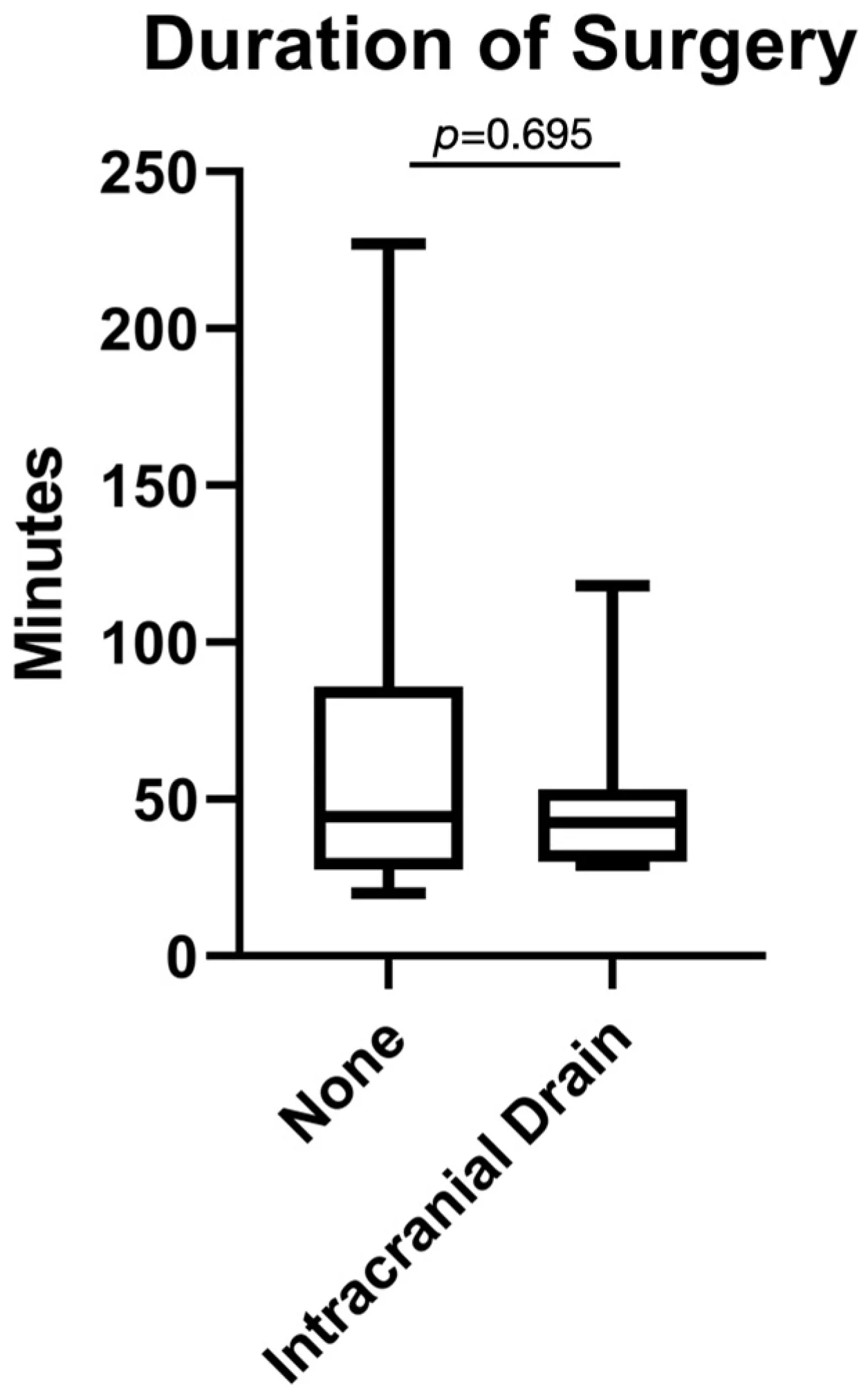
Boxplot illustrating the duration of surgery in patients with and without an intracranial drain.

The duration of surgery is presented in the boxplot in Figure 4. The median operative time was 43 min in patients with an intracranial drain compared to 45 min in those without a drain; however, this difference was not significant (*p* = 0.695).

**Figure 5:**
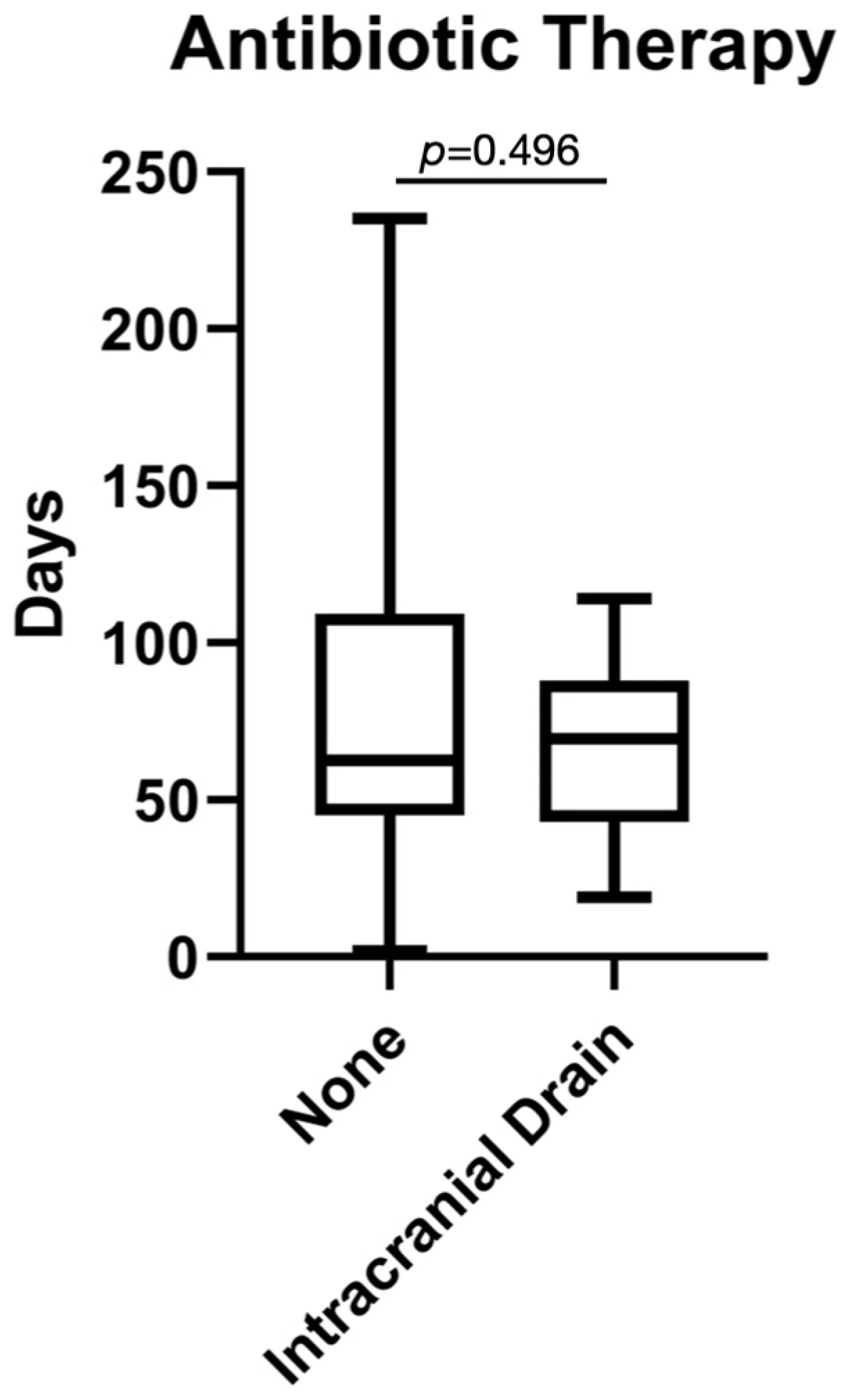
Boxplot illustrating the duration of antibiotic therapy in patients with and without an intracranial drain.

The duration of antibiotic therapy is shown in the boxplot in Figure 5. The median duration was 70 days in patients with an intracranial drain compared to 61 days in those without a drain; however, this difference was not statistically significant (*p* = 0.496).

**Figure 6:**
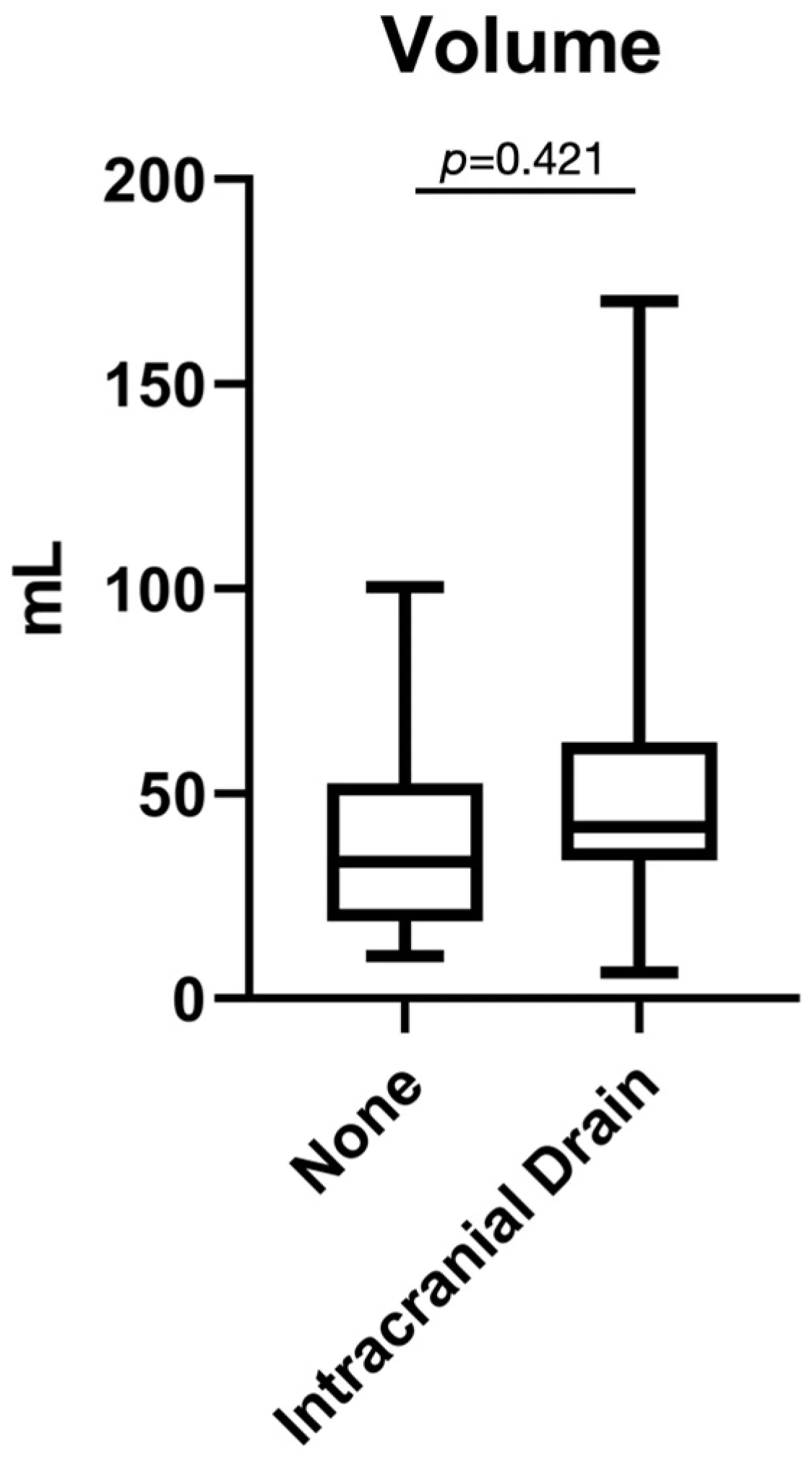
Boxplot illustrating the volume of infectious tissue in milliliters (mL) in patients with and without an intracranial drain.

The volume of the intracranial infection is shown in the boxplot in Figure 6. The median volume was 42 milliliters in patients with an intracranial drain compared to 33 milliliters in those without a drain; however, this difference was not statistically significant (*p* = 0.421).

## 4. Discussion

The treatment of intracranial abscesses and empyemas is, despite progress in diagnostics and therapy, still a neurosurgical and infectiological challenge [13,24]. The combination of antibiotic therapy and surgical intervention remains the standard therapy for abscesses and empyemas [13,25]. The optimal surgical treatment, especially the placement of an intracranial drain, is a highly discussed topic.

The latest guideline of the European Society of Clinical Microbiology and Infectious Diseases recommends early neurosurgical treatment, such as aspiration or excision, if it is technically feasible [13]. The goal of surgical treatment is to reduce the bacterial load, decrease the intracranial pressure, and obtain material for microbiological diagnostics [1,25]. Surgical therapy enables targeted antibiotic treatment [13,15].

The main surgical treatments are stereotactic aspiration, often combined with an intracranial drain, and microsurgical excision [20,25]. Both procedures have improved in recent years due to progress in imaging and navigation systems [18].

Stereotactic aspiration with placement of an intracranial drain is the preferred procedure in many facilities [14,25]. It is minimally invasive and is associated with lower perioperative morbidity [25]. Cai et al. showed that stereotactic aspiration is the most commonly used technique and is associated with good surgical outcomes [20,25]. The advantage of an intracranial drain is that it allows for continuous drainage of pus [18]. It also allows repeated aspiration or lavage of the abscess cavity, especially in larger and more viscous abscesses [18,25]. Surgical treatment is generally the most effective way to reduce intracranial pressure [25]. An intracranial drain makes it possible to obtain repetitive samples for microbiological examination, especially in cases without initial bacterial detection [15]. This enables targeted antibiotic therapy. Current studies show higher success rates in combined surgical and antibiotic treatments [20,25].

However, the rationale for postoperative drain placement remains largely theoretical and is not clearly supported by outcome data. Despite the advantages of aspiration and intracranial drain placement, there are some limitations. A highly discussed issue is the potential need for reoperations, especially in multilocular abscesses [20,24]. In such cases, complete evacuation of pus is difficult. Additionally, the abscess capsule remains intact, which may lead to recurrence [20]. Studies show that the choice of surgical treatment does not significantly influence mortality [14,20]. Another disadvantage is the risk of catheter-associated complications, such as misplacement and infection [14,18].

Microsurgical excision aims to completely remove the abscess along with its capsule [14,20]. This is the preferred procedure in multilocular, encapsulated, or therapy-refractory abscesses [20]. The advantage is a lower recurrence rate, due to complete removal of the infectious tissue [20]. Some studies show comparable success rates compared to aspiration [14,25]. A retrospective study showed a success rate of 84% for aspiration and 76% for patients treated with craniotomy [14,25]. However, surgical excision is a highly invasive procedure and carries a higher risk of neurological deficits, especially in deep or eloquent-location lesions [14]. Therefore, a selective approach is recommended.

The direct comparison of aspiration and excision is controversial. Current studies indicate that both procedures can cause similar clinical improvement and comparable mortality rates [14,20]. The choice of procedure mainly depends on individual factors. Current literature suggests that surgical strategies should be adapted to patient-specific conditions [13,20]. A clear superiority of one procedure has not yet been proven.

Intracranial empyemas differ in their pathophysiology and require more aggressive surgical treatment [26]. Due to their extensive spread, a simple burr-hole is often insufficient. The standard treatment is craniotomy with evacuation of the empyema [26]. Postoperative placement of an intracranial drain is considered inferior to complete surgical clearance [26].

Conservative treatment without surgical intervention may be considered in selected cases, such as high surgical risk or very small abscesses [1,13]. However, current studies strongly recommend surgical drainage in most cases [13,25]. A disadvantage of conservative therapy is the lack of microbiological diagnostics and, consequently, the missed opportunity for targeted antibiotic treatment [15].

As mentioned before, the placement of an intracranial drain is a highly debated topic. Our results show that 50% of our patients with an intracranial drain underwent reoperation, whereas nearly one third of patients without an intracranial drain required reoperation due to progression of infection. This difference could be due to a catheter-associated infection [14,18]. The placement of an intracranial drain leaves an opening for pus discharge, but it also creates a potential pathway for bacteria to enter the infected tissue [18]. Therefore, infection with secondary bacteria is possible. However, in our cohort, the bacterial spectrum did not differ from the initially detected bacteria. Thus, we can reasonably exclude infection caused by secondary bacterial colonization. Another possible reason could be an insufficient discharge of pus during the primary surgery. The surgeries were conducted by different neurosurgeons with varying levels of expertise [20]. Therefore, reoperation rates could be affected by this variability; however, this would also affect the group without an intracranial drain. Consequently, we can assume that this aspect plays only a minor role in the difference in reoperation rates between both groups. Another explanation could be ineffective antibiotic treatment. In our facility, the treatment is initially started with a broad-spectrum antibiotic. Later, after identification of the causative bacteria, the antibiotic regimen is adjusted to a targeted therapy. In our cohort, the initial antibiotic treatment was sufficient for the bacteria detected later. Even if the treatment had been insufficient for the bacteria, this would likely have affected both groups equally.

Our results show that the duration of hospital stay does not differ significantly between the two groups. Nevertheless, patients with an intracranial drain stayed approximately one week longer in the hospital than patients without an intracranial drain. This finding correlates with the higher rate of reoperations in the group with an intracranial drain. If a reoperation is required due to progression of the infectious tissue, the antibiotic treatment must be extended [13,24]. The standard treatment of an intracranial infection consists of at least two weeks of intravenous antibiotic therapy followed by at least four weeks of oral treatment. The intravenous administration must be conducted in the hospital; therefore, a reoperation extends the duration of inpatient care.

Additionally, the placement of an intracranial drain can cause complications, such as intracranial bleeding, secondary infection, misplacement or dislocation of the drain, all of which can negatively affect the clinical well-being of patients [14,18]. These risks must be carefully weighed against the potential benefits of continuous drainage in each individual case. As long as the intracranial drain remains in place, patients require close neurosurgical observation. This monitoring typically includes imaging, neurological assessments, and strict control of infection parameters. Other facilities, such as departments of internal medicine or geriatric medicine, which are also capable of administering intravenous antibiotic therapy, are often not equipped to manage patients with intracranial drains. This limitation is mainly due to the need for specialized expertise and equipment in handling neurosurgical devices. Therefore, a prolonged stay in a neurosurgical unit is frequently necessary.

The placement of an intracranial drain can also influence the duration of surgery. A longer surgical procedure is generally associated with a higher risk of complications, such as infections, wound healing disorders, thrombosis, embolism, or cardiac events [27]. These risks are particularly relevant in elderly or multimorbid patients [27]. Our results show that the duration of surgery was not significantly altered. Therefore, we can assume that the placement of an intracranial drain is a relatively safe procedure that does not substantially prolong surgical time. However, it may also indicate that surgeons who place an intracranial drain tend to complete the procedure more quickly, potentially without sufficiently evacuating all pus. The main purpose of an intracranial drain is to allow continued discharge of pus after completion of surgery. The higher median duration of surgery in the group without an intracranial drain may be explained by a more thorough resection or evacuation of infectious tissue. Without the option of postoperative drainage, some surgeons may adopt a more radical and time-consuming surgical technique to ensure adequate removal of infected material. This approach may reduce the need for further interventions but increases operative burden.

The main purpose of an intracranial drain is to reduce the bacterial load by facilitating continuous pus discharge and thereby accelerating the healing process [1,25]. However, our results show that the duration of antibiotic treatment did not significantly differ between the two groups. This may be explained by the more radical surgical approach in patients without an intracranial drain. Following extensive resection or evacuation, the bacterial load is reduced more effectively, which may accelerate recovery, whereas an intracranial drain provides gradual but continuous drainage. Both strategies may ultimately lead to comparable clinical outcomes despite different mechanisms. Another possible explanation could be secondary infections associated with the placement of an intracranial drain, as it leaves an opening through which bacteria can enter intracranially. Additionally, intracranial drains are often placed in cases with a large volume of intracranial pus [25]. Surgeons typically tend to use drains in larger abscesses. However, our results show no significant difference in abscess volume between the two groups. This indicates that the duration of antibiotic treatment was not primarily influenced by the volume of infection.

From an economic point of view, the expenses for an intracranial drain need to be considered. Our results show that the placement of an intracranial drain does not benefit the clinical outcome. Therefore, the additional costs for an intracranial drain, such as a trocar, the catheter, the reservoir, the stitches, longer operation, and anesthesia time, could be saved.

### Limitations

We acknowledge that our study has several limitations. First, it is a retrospective study including a limited number of patients, which inherently introduces certain methodological shortcomings. Second, the surgeries were performed by different neurosurgeons, leading to intraoperative variability in surgical techniques and approaches. Although surgical strategies are generally similar within our institution, individual preferences may influence clinical outcomes. Third, the inclusion of four patients with an intracranial empyema might influence the statistical differences in abscess patients. Lastly, the volumetric measurements were performed by a single neurosurgeon and only once, which reduces both intra- and inter-rater reliability.

## 5. Conclusions

The treatment of intracranial abscesses and empyemas has made significant progress in recent years. Stereotactic aspiration of intracranial abscesses is currently the preferred treatment due to its minimally invasive nature and favorable clinical outcomes. Nevertheless, microsurgical excision of infectious tissue remains an important alternative approach in selected cases. However, the placement of an intracranial drain remains a highly debated issue with a heterogeneous body of evidence. Our results show no significant differences between patients treated with and without an intracranial drain. Considering our limitations, we would not recommend the routine placement of an intracranial drain. Further prospective studies are required to evaluate optimal drainage strategies, assess the role of intracavitary application, and identify prognostic factors. Such efforts are essential to develop evidence-based, individualized treatment concepts for patients with intracranial abscesses and empyemas.

## Figures and Tables

**Table 1:** Comparison of groups with and without intracranial drain.

	Intracranial Drain	None	*p*-Value
**Overall**	8 (100%)	28 (100%)	
**Median Age [years] (IQR)**	47 (40–63)	60 (42–68)	0.378
**Sex**			1.000
Female	2 (25.0%)	9 (32.1%)	
Male	6 (75.0%)	19 (67.9%)	
**Median Hospital Stay [days] (IQR)**	29 (18–35)	18 (14–32)	0.145
**Median Duration of Surgery [min] (IQR)**	43 (30–53)	45 (28–121)	0.695
**Median Antibiotic Therapy [days] (IQR)**	70 (43–88)	61 (46–110)	0.496
**Median Volume [mL] (IQR)**	42 (34–63)	33 (19–53)	0.421
**Median Days till Reoperation (IQR)**	12 (9–12)	11 (6–15)	0.641
**mRS before Surgery**	3 (1–4)	3 (1–4)	0.641
**mRS after Surgery**	1.5 (0–3)	2 (1–3)	0.421
**Reoperation**	4 (50.0%)	9 (32.1%)	0.422
Readmission	0 (0%)	4 (14.3%)	0.555
**Approach**			0.397
Craniotomy	1 (12.5%)	9 (32.1%)	
Burr-Hole	7 (87.5%)	19 (67.9%)	

IQR: interquartile range; mRS: Modified Rankin Scale.

**Table 2:** Comparison of approach to reoperation rate.

	Craniotomy	Burr-Hole	*p*-Value
**Overall**	10 (100%)	26 (100%)	
**Reoperation**	4 (40%)	9 (34.6%)	1.000

## Data Availability

All data generated or analyzed during this study are included in this published article.

## Abbreviations

The following abbreviations are used in this manuscript:

IQR	Interquartile Range
mL	Milliliter
MRI	Magnetic Resonance Imaging
mRS	Modified Rankin Scale
